# Kyphosis in spinal tuberculosis – Prevention and correction

**DOI:** 10.4103/0019-5413.61893

**Published:** 2010

**Authors:** Anil K Jain, Ish Kumar Dhammi, Saurabh Jain, Puneet Mishra

**Affiliations:** University College of Medical Sciences and GTB Hospital, Delhi-110 095, India

**Keywords:** Kyphotic deformity, late onset paraplegia, TB spine, kyphus correction, extrapleural anterolateral approach

## Abstract

Spinal deformity and paraplegia/quadriplegia are the most common complications of tuberculosis (TB) of spine. TB of dorsal spine almost always produces kyphosis while cervical and lumbar spine shows reversal of lordosis to begin with followed by kyphosis. kyphosis continues to increase in adults when patients are treated nonoperatively or by surgical decompression. In children, kyphosis continues to increase even after healing of the tubercular disease. The residual, healed kyphosis on a long follow-up produces painful costopelvic impingement, reduced vital capacity and eventually respiratory complications; spinal canal stenosis proximal to the kyphosis and paraplegia with healed disease, thus affecting the quality and span of life. These complications can be avoided by early diagnosis of tubercular spine lesion to heal with minimal or no kyphosis. When tubercular lesion reports with kyphosis of more than 50° or is likely to progress further, they should be undertaken for kyphus correction. The sequential steps of kyphosis correction include anterior decompression and corpectomy, posterior column shortening, posterior instrumentation, anterior bone grafting and posterior fusion. During the procedure, the spinal cord should be kept under vision so that it should not elongate. Internal kyphectomy (gibbectomy) is a preferred treatment for late onset paraplegia with severe healed kyphosis.

## INTRODUCTION

**Figure 1:**
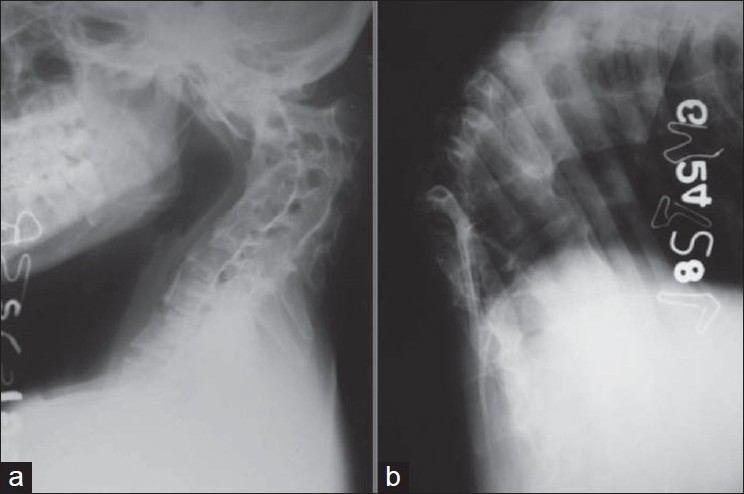
Plain X-ray (lateral view) of cervical spine (a) and dorsal spine (b) shows a severe healed kyphosis of upper cervical spine and dorsolumbar spine as a sequelae of TB spine

Neurological complications (paraplegia or quadriplegia) and spinal deformity are the most dreaded complications of tuberculosis of spine.^1^ Neurological complications develop in the active or healed stage of the disease. The sequelae of these two complications affect the quality and span of life. Almost all tuberculosis of spine, even if they are treated well, leave behind some amount of kyphosis in different segments of spine [Figure 1]. Persistent spinal deformity affects the biomechanics of all segments of the spine. The life expectancy of human beings has increased globally. If deformity is moderate to severe, these patients report 10–20yrs later with the clinical problems related to persistent spinal deformity and paraplegia with the healed disease.

The objective of treatment of spinal tuberculosis in the preantibiotic era used to be, to achieve bacteriological quiescence of spinal tubercular lesion, probably by natural immunity. With the advent of effective chemotherapy the objective of the treatment has changed to healing of spinal TB with residual spinal deformity. Development in the field of imaging has allowed us to diagnose TB of spine in less advanced stage of the disease. With better operating theatre (OT) facilities, intensive care units (ICU), spinal instrumentation and diagnostics we should aim to achieve healed status of disease with minimal or no spinal deformity. The issues which need careful discussion are identification of adult patients in whom kyphosis will increase on treatment producing severe deformity, children in whom kyphus should be taken for correction and to reach a consensus on treatment when patient presents with severe healed kyphosis with or without neural deficit. This review article is presented to analyze the pathology of kyphosis in spinal TB and discuss strategies to prevent/correct post-tubercular kyphotic deformities.

## PATHOLOGY OF KYPHOTIC DEFORMITY IN SPINAL TUBERCULOSIS

The paradiscal regions of vertebrae are affected in 98% of TB spine lesion. The tuberculous lesion starts as a paradiscal inflammation. Gradually as disease progresses the vertebral end plates become structurally weak and intervertebral disc starts ballooning/herniating into the diseased vertebral body. It is seen on plain X-rays as reduced disc height while truly on MRI the disc maintains its height and hydration for quite some time^2^ and gradually herniates into the diseased vertebral bodies. Since the line of weight transmission in thoracic spine is in the anterior half of vertebral bodies, the vertebral body loses more anterior height than posterior. Thoracic kyphosis increases and gradually an angular kyphosis appears. The severity of kyphosis depends on the number of vertebral bodies affected, severity of loss of anterior vertebral body height and segment of the spine affected. A case of dorsal spine or dorsolumbar spinal tuberculosis with three or more vertebral body affection is more likely to develop moderate to severe kyphotic deformity. In cervical and lumbar spine, the line of weight transmission is in posterior half of vertebral bodies hence it causes first obliteration of natural cervical and/or lumbar lordosis and later on kyphosis starts appearing. By the time a kyphosis appears in the spine the disease is already in about three to four months of pathogenesis of the tuberculosis of spine. About 95% shows a clinically detectable kyphosis or reversal of normal lordosis, when patient reports for specialized treatment in developing countries.^3^

## BEHAVIOR OF KYPHOSIS IN ADULTS

The vertebral column is structurally weak as a result of being affected by the disease process. Normal stresses and strains of daily activities lead to pathological fracture in the diseased vertebrae and it can collapse into a kyphosis.

Once the diagnosis of TB spine is made and patient is put on ambulant chemotherapy, kyphosis continues to grow despite being treated. However, the progression of kyphosis can be minimized by prescribing suitable braces. Patients treated nonoperatively have an average increase of 15° in deformity and three to five per cent end up with a deformity greater than 60°.^3^,^4^ Kyphosis also continues to grow in a TB spine lesion which is surgically decompressed and bridged by bone graft. The bone graft is most weak on the day it is implanted. As a result, graft related complications such as graft slippage and breakage will give rise to progression of kyphosis.^4^–^6^ Kyphotic deformity increases more following surgical decompression in comparison to non-operative treatment. After surgical decompression the increase in deformity is more in patients who have a long segment disease necessitating a long bone graft in a dorsal or dorsolumbar spine. Upadhyay *et al*, observed increase in kyphosis for only six months following a decompression surgery.^7^ Kyphosis, once healed, with osseous fusion does not grow alarmingly in adults in later life. However when the lesion heals with fibrous or fibro-osseous healing it may progresses further.

## BEHAVIOR OF KYPHOSIS IN CHILDREN

The TB spine lesion in children causes more destruction as most of the vertebral bodies are cartilaginous. Even when a TB lesion heals by nonoperative treatment kyphosis continues to increase with growth. The anterior growth potential of the vertebral body is either destroyed because of disease itself or because of surgical excision of disease focus or may be due to the alteration in the growth resulting from the effect of biomechanical forces on the growth plate of both the fusion mass and the vertebral segments within the kyphotic region.^8^ Unabated posterior growth may contribute to increase in the kyphotic deformity. Upadhyay *et al*. reported two series on kyphosis behavior after a long follow-up of debridement surgery in children in comparison to adults^8^ and radical resection and posterior arthrodesis in children in comparison to adults.^9^ The mean follow-up was 19.6 yrs and 15 yrs respectively. He did not notice any evidence of disproportionate posterior spinal growth contributing to the progression of kyphosis in children.^8^,^9^ However, these series were a part of MRC multicentric trials which did not include a severe affection. Schulitz *et al*. analyzed changes of spinal deformity during growth period with regard to the operations performed for spinal TB in children. 117 children were analyzed 5–10 yrs after the operation. The operative procedures were anterior radical excision and fusion, posterior fusion, combined anterior and posterior fusion and anterior debridement without fusion. The patient treated with radical resection and anterior fusion showed the worst results with respect to progression of kyphosis particularly when the lesion was in thoracic spine and several segments were involved. Combined fusion and only anterior debridement has least progression of kyphosis. This suggests that posterior elements do contribute to growth and as far as possible anterior radical resection should be avoided in children.^10^

**Figure 2:**
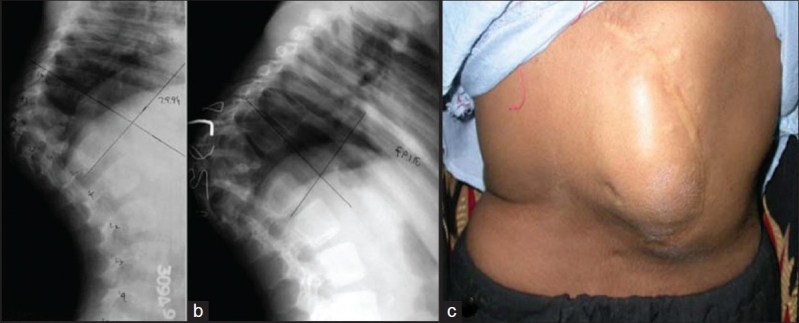
Lateral X-ray of a dorsolumbar spine (a) shows a severe kyphosis secondary to childhood tuberculosis spine with complete destruction of D9–11. This kyphosis is expected to grow with growth. Lateral X-ray of the same patients (b) 2½ years later shows progression of kyphosis in spite of successful posterior spinal fusion. Clinical photograph (c) of the same patient shows deformity and scar of posterior fusion

The TB lesion which has severe anterior vertebral body loss with moderate to severe kyphosis, the spine keeps collapsing at the apex of kyphosis till a proximal vertebral body rests stably on a distal vertebral body. If this does not happen, in view of increase in kyphosis, the proximal vertebral body gets rotated by 90° and the anterior border of proximal vertebral body then rests on the distal vertebral body. As a result, any further growth which takes place is added to kyphosis which keeps increasing exponentially. Rajasekaran has studied the natural history of kyphosis in children.^11^–^13^ During growth spurt in a child, 44% cases encounter improvement in kyphosis while 17% show no changes in kyphotic angle. About 39% cases have progression of kyphosis with growth; 10% will have exponential progression of kyphosis to produce a severe kyphosis of more than 90°.^11^–^13^ The risk factors for exponential increase in kyphosis in children include age less than seven years at the time of disease, thoracolumbar involvement, loss of more than two vertebral bodies and presence of two or more spine at risk signs.^11^ It is these children [Figure 2] who need to be identified and kept under observation to be surgically treated to prevent the sequalae of severe kyphosis.^11^–^13^

## SEQUALAE OF SEVERE KYPHOSIS

**Figure 3:**
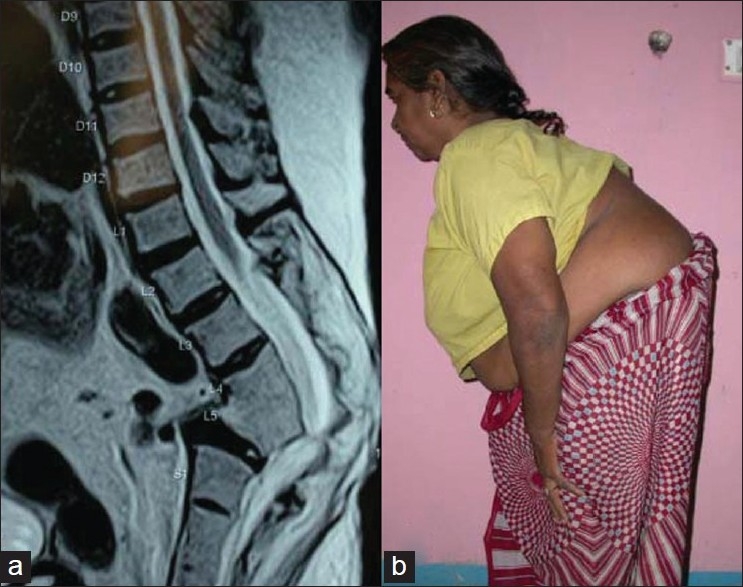
T_2_ weighted MRI of lumbosacral spine (a) shows a healed kyphosis with lumbar canal stenosis. Clinical photograph (b) of the same patient shows the posture with forward bending due to severe neurogenic claudication

Kyphosis on a long follow-up affects the biomechanics of the spine and body. The proximal and distal segments of the spine compensate by creating reverse deformity as a result of kyphosis. The consequent degenerative process will produce back pain and/or radiculopathies. If a dorsal kyphosis is more than 60°, the spinal cord undergoes changes as a result of repeated stretching over internal salient and may develop clinical signs of upper motor neuron deficit which has worse prognosis in comparison to paraplegia with active disease.^1^,^3^ Acute angular kyphosis at lumbosacral junction or upper cervical spine may produce neural deficit early. The lumbar or lumbosacral kyphosis may produce a compensatory hyperlordosis proximally and distal to healed lesion. On a long follow-up they develop evidence of severe lumbar canal stenosis.^14^ These morphological changes occur in both fusion mass and uninvolved level [Figure 3].^12^–^14^ The capacity of chest cavity and inturn vital capacity is reduced. The costal margins come closer to iliac crest and diaphragm gets pushed into the chest cavity, reducing the vital capacity further. Ventilatory failure may develop after a gap of many years in patients with a severe thoracic kyphosis due to tuberculosis. Gradual impingement of costal margins over iliac crest gets painful.^3^

## TREATMENT OF SPINAL TUBERCULOSIS WITH KYPHOSIS

The development of severe kyphosis and its sequelae can be prevented by

- diagnosing TB spine early before a kyphosis develops,
- identifying those TB spines which are likely to have severe kyphosis at the end of the treatment and progression with growth,
- by correcting angular kyphosis in active stage of disease.

### 1. Early diagnosis of TB spine (well before deformity develops)

TB spine is a slowly developing disease and it takes three-four months in pathogenesis before kyphosis starts appearing. During these three-four months these patients present repeatedly with persistent low back pain with or without constitutional symptoms. In the initial stages X-rays of these patients, may not have any radiological finding. Such patients with back pain should be kept under observation in an endemic region for tuberculosis and if sequential X-rays, with a gap of four to six weeks, show reduction of disc height they should be subjected to MRI. On MRI they can be confidently labeled as TB spine if the vertebrae show low signal in T1WI weighted and hyper-intense signal in T2WI, suggestive of inflammation with a septate pre and para vertebral abscess and a contiguous vertebral body involvement with preserved intervertebral disc. In the absence of such finding, a CT guided core needle biopsy will help reach an early diagnosis. The TB spine lesions, when diagnosed in predestructive stage of disease (inflammatory stage), may heal by uninterrupted ambulatory antitubercular chemotherapy with no sequelae of kyphosis.^2^

### 2. Patients report with classical disease

The objective of treatment should be to treat these patients in a manner that kyphosis does not progress on treatment. The case should be evaluated by imaging adequately to rule out concomitant posterior column involvement along with vertebral body disease. Such cases should be surgically stabilized. The kyphosis which is likely to progress on treatment needs to be kept under observation and for need to be corrected/ stabilized in active stages of the disease.

The progression of kyphosis depends on number of vertebral involvement, initial vertebral body loss and segment of spine affected.

One vertebral body height loss gives rise to about 30°–35° kyphosis. The acceptable kyphosis may vary with different segment of spine. Tuli was of the opinion that if patients developed 60° or more kyphosis at dorsal or dorsolumbar spine they were likely to develop late onset paraplegia.^3^ Now, with increased life expectancy, these patients are likely to live longer and a localized kyphosis of 50°–60° may be disabling due to biomechanical stresses on proximal and distal segments of spine even though late onset paraplegia may not develop.

The kyphosis continue to increase on non-operative treatment. Rajasekaran and Soundarapandian^6^ suggested a formula to predict final kyphotic deformity in adult patients of TB spine; Y=a+bx, where Y is the final angle, “a” and “b” are the constant 5.5 and 30.5 respectively and x is the initial loss of height of vertebral body. Jain *et al*. observed the behavior of kyphotic angle in the spinal tuberculosis in 70 adult cases of spinal tuberculosis and observed that prediction of kyphosis is possible with this formula. However, the angle could be predicted by ±10°, hence any patient with initial vertebral body loss of 1.5 vertebral body height in dorsal and dorsolumbar spine will develop kyphosis of 50°±10°.^16^

The kyphosis continues to progress after surgical decompression.^17^ Graft is able to provide sufficient stability and structural support in only 41% of patients with a short defect. Graft related complications occur when length of graft spans two vertebral body heights (four to five cm). In all such instances additional support in the form of extended bed rest, plaster jacket or instrumented stabilization is indicated.^5^ All patients of TB spine, likely to heal with more than 50° kyphosis need a closer observation and, if indicated, kyphus correction surgery.

In adult patients, three or more vertebral body affection with initial vertebral body loss of one to 1.5 vertebral body height in a dorsal or dorsolumbar spine, need to be taken up for kyphosis correction. Children younger than seven years of age with three or more vertebral body affection in dorsal or dorsolumbar spine with two or more spine at risk segment will have a progressive kyphosis with growth; hence those should be undertaken for correction of kyphosis and stabilization of spine in active stage of disease. The radiological signs of spine at risk are; a) subluxation or dislocation of the facet joint at the apex of the kyphus b) presence of retropulsion c) translation of the vertebral column in coronal plane d) posterior toppling sign (i.e. if a line drawn along the anterior border of the distal healthy vertebra in an upward direction that meets the upper half of proximal healthy vertebra then it has chances of progressive kyphosis.^13^ Hence these cases should undergo kyphosis correction.

### 3. Kyphosis correction in active disease

All kyphosis cases which report for the first time with moderate to severe kyphosis of 50° of more, may be taken up for kyphosis correction surgery. Lots of studies are available where outcome of kyphosis is described. However, only few studies cover the broad principles of kyphosis correction in spinal tuberculosis.^18^–^21^ Most of the studies describe one stage or two stage surgery to decompress the spinal cord and stabilize the spine with the objective to prevent post treatment deterioration of kyphosis.

Many studies have described anterior or posterior instrumentations to correct kyphosis or prevent on treatment deterioration of kyphosis. Unfortunately the variables to analyze kyphosis were not described by most of them.^18^ In cumulative cases with anterior instrumentation (n=635), mean 25° kyphosis was brought to 9° post-operatively and final kyphosis remained 11° while in posterior instrumented group (n=369) mean kyphosis of 40° was corrected to 18° and which finally healed by 22°. It seems these surgeries were primarily done for stabilization of spine to prevent progression of kyphosis and not for correction of kyphosis.^17^

Spinal TB has an angular kyphosis produced by diseased vertebra. Usually in TB spine 3 or more vertebral bodies are diseased and have lost anterior body height. A few more vertebral bodies may be inflamed. The spinal cord may be compressed anteriorly, with disc, granulation tissue and pus and meninges may be inflamed, hence more vulnerable for neural deterioration. Epidural blood vessels and intercostal and anterior spinal arteries may be thrombosed.

The issues involved in kyphosis correction include:-

1. The spinal tuberculosis lesion has a retropulsion of disc, granulation tissue and bony sequestrum. Correction of the kyphosis without opening the disease area and without anterior decompression will produce more prominent spinal cord indentation by retropulsed fragment (internal salient) and consequently neural deficit. Hence internal salient should be removed by anterior debridement and corpectomy.^22^
2. Moderate to severe kyphosis which needs correction is a long standing problem. The vertebral column is shortened anteriorly and spinal cord has adjusted in kyphosis to a shortened length hence abrupt correction of kyphosis will produce lengthening of anterior column. This will stretch the spinal cord with consequent neural deficit. Thus it is desirable to shorten the vertebral column posteriorly.^22^
3. Once anterior corpectomy and posterior column shortening is done, the spine becomes grossly unstable. Hence the spine needs instrumented stabilization with anterior gap grafting and posterior fusion.^22^
4. The anterior implant may not have a strong hold and span of instrumentation may be too long to withstand deforming forces, hence posterior instrumentation may be desirable.
5. The kyphosis correction in TB spine involves anterior corpectomy to achieve anterior decompression, shortening of posterior column, posterior instrumentation and anterior and posterior bone grafting. Ideally both steps should be undertaken in the same sitting and sequentialy.^23^
6. In whole procedure the spinal cord should be kept under vision and correction of kyphosis should be so gradual that the spinal cord is not elongated.

The reported methods of kyphosis correction in spinal TB are: -

- Single stage transpedicular approach.
- Single or two-staged anterior decompression with bone grafting followed by correction of kyphosis and posterior instrumentation or vice versa where posterior instrumentation was done first followed by anterior surgery.
- Single stage kyphosis correction by extra pleural anterolateral approach.

#### a. Single stage transpedicular approach

**Figure 4:**
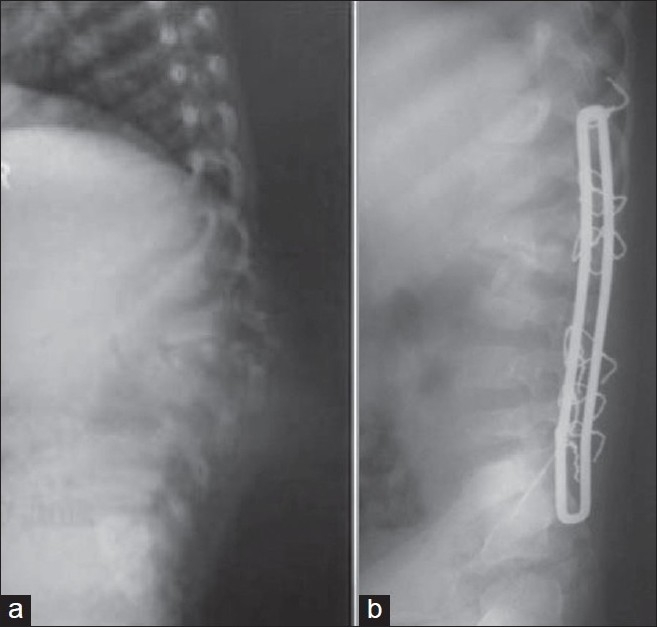
Lateral X-ray of lumbosacral spine (a) in a four-year-old child shows TB of L1–L3 with kyphosis of 40°. Lateral X-ray of same patient (b) where lumbar kyphosis is corrected with pedicle subtraction and Hartshill fixation. The kyphosis is corrected to 10°

The anterior decompression was performed by posterior midline exposure through the pedicle of apex vertebra, followed by posterior stabilization with two vertebrae on each side of dorsal spine and one in lumbar spine. This procedure was performed to prevent postsurger y progression of kyphosis.^21^ The author reported only 3.4° loss of correction till complete healing of disease. The objective in this series was prevention of postsurgery deterioration of kyphosis, as seven cases had two vertebral body disease and three had one vertebral body disease.^21^ Most notable series as reported by Laheri *et al*.^23^ where 28 patients of post tubercular kyphosis (mean 64.3°) were operated. The patients were put in prone position. Posterolateral retropleural approach was used. A midline incision was made centering at kyphosis. A costotransversectomy and excision of pedicle was carried out at the apex of the kyphosis. The abscess was drained, granulation tissue and bony sequestrum were debrided and spinal cord was decompressed. All bony tissues and soft tissues preventing the correction of kyphosis were removed. Segmental spinal instrumentation using Hartshill rectangle with sublaminar wiring or pedicle instrumentation was done [Figure 4] and kyphosis is gradually corrected. The author was careful in achieving gradual kyphosis correction to avoid spinal cord stretch. The anterior defect was subsequently grafted.^23^

Lee *el. al*. has performed single stage transpedicular decompression and posterior instrumentation in active TB spine lesion where bone distruction was less (n=10) and compared with anterior decompression fusion and anterior instrumentation (n=7). This was not aimed at kyphosis correction and mean pre-operative kyphosis was corrected from 18°–20° to 14°–16°.^24^ Gokce *et al*. reported correction of sagittal balance in 12 TB spine cases with kyphosis by posterior closing wedge osteotomy with posterior instrumented fusion.^25^ The patients were taken in prone position and posterior approach was used. The level to be instrumented were exposed to the tips of transverse process at lumbar and to costal attachment at thoracic region. The pedicle screws are inserted and temporary stabilization was done. Laminectomy and posterior decompression of the apex vertebral was done.^25^ At the apex of the deformity soft tissue on the lateral wall of the pedicle and vertebral body to be removed are dissected bluntly and elevated on both sides. The cancellous bones were curetted until only the residual bony cortex remained. The adjacent end plates and posterior part of the vertebral body attached to the posterior longitudinal ligament were removed. The wedge shaped portion of the cortical bone at the vertebral body was removed. Gradual closure of the spine was done with closing dorsally based osteotomy site. The author reports correction of kyphosis from 51.1° to 23° in lower dorsal and dorsolumbar spine.

Transpedicular decancellation osteotomy^26^,^27^ has also been described for healed post tubercular kyphosis at dorsolumbar spine. Here again pedicle screws are placed two vertebrae above and two below. The laminectomy of the apex vertebrae is done. The egg shell decancellation of the vertebral body (kyphotic mass) was done through pedicle preserving medial wall of pedicle. Temporary rod stabilization on one side is done. Once decancellation is done or adjacent discs are removed from the adjacent side of the vertebral bodies, the rods are contoured and placed bilaterally. The reduction is carried out via sequential compression on the rods until posterior elements starts touching. The thecal sac is constantly kept under observation during the reduction.

**Figure 5:**
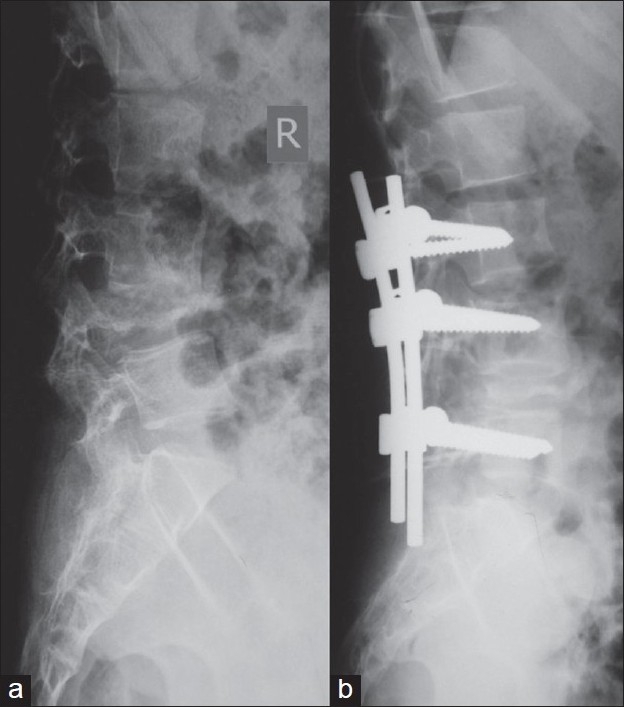
Lateral X-ray of LS spine (a) of a 15-year-old patient with TB spine lesion of L4–5 with kyphosis of 25°. The lumbar kyphosis was corrected by pedicle subtraction osteotomy and fixed with pedicle screw (b) and kyphus is corrected to 0°

This method has been reported with a successful correction of kyphosis in dorsolumbar and lumbar kyphosis with mean pre-operative kyphosis correction from 29.9° to 12.2°^26^ and mean pre operative kyphosis from 58.8° to 13.7° respectively in two series [Figure 5].^27^

#### b. Single or two-stage anterior and posterior surgery

Here the correction of kyphosis could be achieved by anterior decompression followed by posterior instrumentation. After anterior decompression by transthoracic transpleural or retroperitoneal approach the gap may be filled by the bone graft. 2–3 weeks later the posterior instrumentation can be performed.

Moon performed posterior instrumentation first followed by transthoracic decompression and anterior bone grafting two weeks later in earlier part of the study. However, later on he performed both in the same sitting.^20^ The mean preoperative, immediate postoperative kyphosis and final kyphosis was 37°, 16° and 18° respectively. These cases did not have severe kyphosis. He primarily aimed at prevention of deterioration of kyphosis and not for kyphosis correction.

Louw reported a series of 19 patients of TB spine of dorsal and dorsolumbar region with neural deficit. He performed transthoracic transpleural anterior decompression and vascularized rib grafting followed, during the same procedure (n=13) or 2 weeks later (n=6) by shortening of posterior column by multilevel posterior osteotomy, instrumentation and fusion. Thus overall anterior column length was not altered and kyphosis was corrected with anterior graft acting as a pivot. The mean preoperative kyphosis could be corrected from 56 to 27°.^28^

#### c. Single stage kyphosis correction by extrapleural anterolateral approach

Here kyphus correction is done by extrapleural anterolateral approach with patient in a lateral position. The author believe that ideal procedure for kyphus correction in spinal tuberculosis is anterior decompression and corpectomy followed by posterior column shortening, instrumented stabilization and reconstruction of anterior gap by bone graft sequentially in one stage. The patient is placed in a lateral position and a T-shaped posterior incision of about 14–15 cm is given with the centre of lesion in the apex of the kyphosis and the transverse incision about 8 cm from the midline and perpendicular to it at the apex of kyphosis on left side. Standard extra pleural anterolateral decompression is performed after removing posterior 6–8 cm of 3 ribs at the apex of the lesion. The diseased apex vertebral body is removed as far as possible to the opposite side. The anterior wound is now packed. The posterior para spinal exposure is done to expose three segments on either side of apex vertebrae. The space for passing sublaminar wire is created and sublaminar wires are passed. Now Hartshill rectangle is selected and a prebend shape is given. The spinous process, laminae, pedicles of the vertebrae at apex of kyphosis is removed. The posterior part of the rib attached at the apex vertebrae on right side is also removed. At this stage the spinal cord has no bone around it. The sublaminar wires are tightened first distally and then proximally to gradually correct the kyphosis [Figure 6]. At all times spinal cord is kept under vision and correction is carefully done to ensure that spinal cord is not elongated. Once correction is achieved the anterior grafting is done from a graft taken from the iliac crest and ribs are used as bone graft for posterior spinal fusion. The wakeup test should be done for all those who have no neural deficit.^22^

**Figure 6A:**
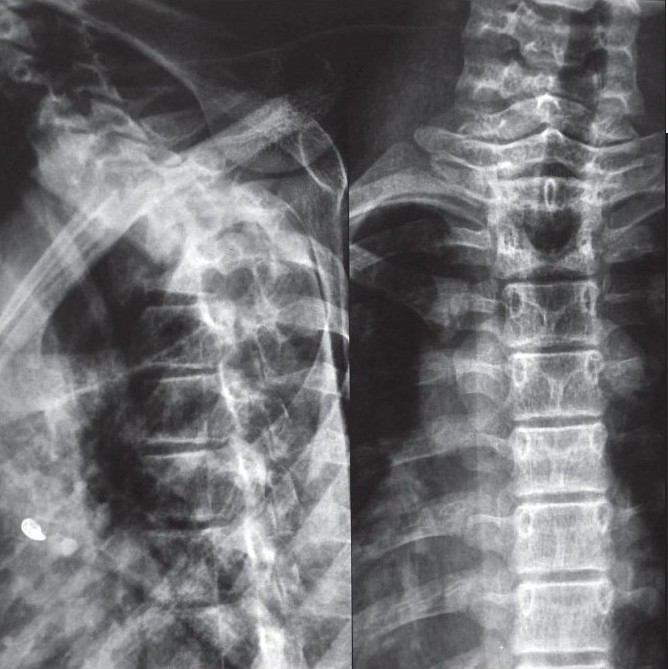
Lateral and anteroposterior X-rays of upper dorsal spine lesion show kyphosis of 80° of upper dorsal spine. Anteroposterior view shows end on appearance of D 1, 2, 3 with crowding of ribs

**Figure 6B:**
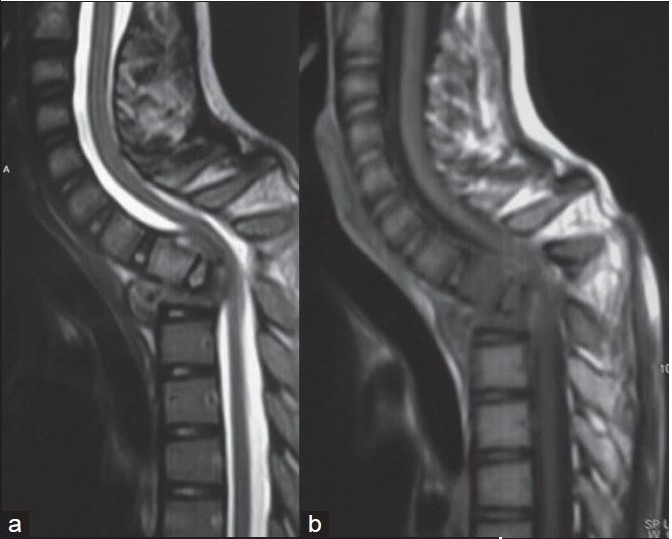
T2WI (a) and T1WI (b) of same patient shows complete destruction of D2,3 with tilting of D1 causing indentation on spinal cord with pre vertebral, paravertebral and intraspinal cord compression

**Figure 6C:**
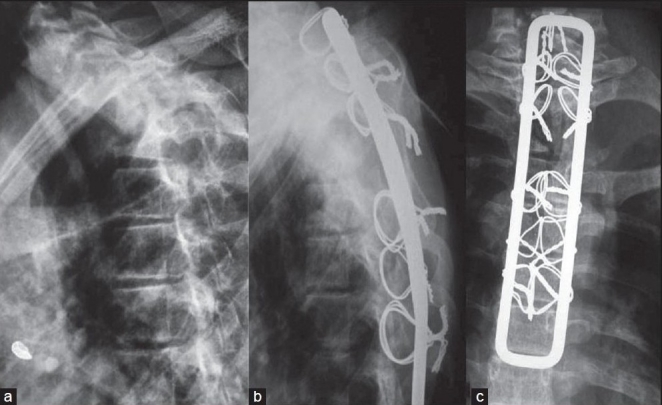
The preoperative lateral X-ray (a) of the same patient shows preoperative kyphosis of 80°. The anterior decompression with kyphus correction and Hartshill fixation was done with extra pleural anterolateral approach. Follow-up lateral X-ray (b) shows corrected kyphosis to 40° and AP X-ray (c) shows resection of second, third and fourth ribs and cortico cancellous grafting in place with Hartshill instrumentation *in situ*

The advantages of this procedure are:

- Patient is in lateral position; hence remains stable all the time and there is no need of temporary stabilization.
- Chest cavity and retroperitoneal exposure is not needed, hence it is less morbid.
- It provides simultaneous exposure of anterior and posterior columns of the spine, there by allowing opportunity to work anterior and posterior procedure simultaneously.
- Kyphosis from D2 to L2 can be corrected by this approach.

### 4. Kyphosis correction in healed lesion

Severe kyphosis is not mere disfigurement. It produces progressive restriction of pulmonary functions. In one study, out of 23 patients of severe kyphosis, 11 had more than 50% restriction of pulmonary functions, while 10 had between 25–50% and 2 had mild restriction of pulmonary functions.^29^ Correction of such severe kyphosis of long standing is a technically demanding surgery with higher risk of neurologic injury. Yau has performed a multi-staged surgery where anterior osteotomy and decompression was performed after fitting a halo pelvic distracter. In second stage posterior osteotomy and fusion was done. As a third stage, again anterior fusion was done. The patient was maintained in halo pelvic distractor till sound fusion was achieved. The mean preoperative kyphosis in this series was 115.5° and the correction obtained was 28.3°. 3 patients out of 29 died. The author concluded that it may be a relatively small reward for such a major undertaking. Such treatment should be instituted where deformity is severe with active disease and paraplegia or death from chest complication is imminent.^29^ Yau and many other authors later on also believed that for patients with healed disease in whom danger of paraplegia and rapid progression of deformity are less, the hazard of deformity correction outweighs the gain hence it should not be carried out for cosmetic reasons only.^29^,^30^ Even for late onset paraplegia the surgeon should not be too ambitious to correct deformity and the patient should be forewarned about the risk of neural deterioration and life risk. However, correction of healed kyphosis of lower dorsal and dorsolumbar spine by transpedicular decancellation osteotomy has recenty been reported.^27^

### 5. Late onset paraplegia or paraplegia with healed disease

Here, patients report with long standing severe kyphosis with a history of being treated for spinal TB, 10 or more years ago and now present with signs of upper motor neuron spinal cord injury or paraplegia. The cause may be a reactivation of old healed lesion at kyphus or intrinsic changes in spinal cord due to continued stretch on internal salient.

Anterior decompression and fusion is advocated in all such cases. Here internal salient is removed either by transthoracic transpleural anterior approach or by the extrapleural anterolateral approach. The internal kyphectomy allows spinal cord to transpose anteriorly. The response to anterior decompression is faster, better and safe in patients with active disease. While in patients with healed disease the anterior decompression is technically more difficult and the recovery less satisfactory.^30^ Complications such as neural deterioration (transient or permanent) and CSF fistula have been reported^31^ and patient should be warned before the surgery about the possibility of neural deterioration.

**Figure 7:**
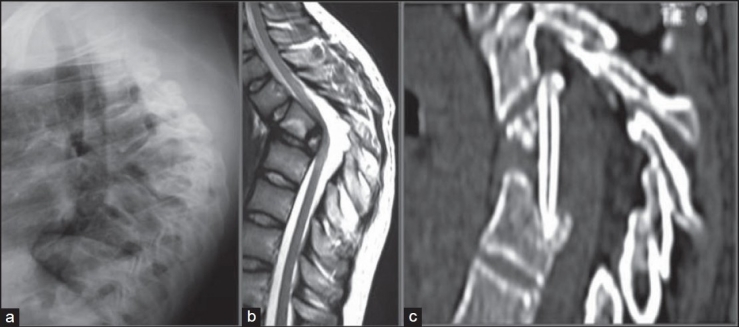
Lateral X-ray of dorsal spine (a) in a 20-year-old boy with late onset paraplegia: The patient had TB spine at the age of five years. D5–7 has become a bone block with 90° kyphosis and child has reported with mild motor weakness with spasticity. T2 WI mid sagittal MRI (b) scan shows kyphotic spine with internal salient indenting and atrophied spinal cord. The reconstructed mid saggital CT image (c) after internal kyphectomy: The picture shows spacious spinal cord with bridge tricortical graft *in situ*. The kyphus was not corrected and the patient showed significant neural improvement

Since these patients already have a compromised pulmonary function extra pleural anterolateral or costotransversectomy approach is advocated^32^. The other advantages of this approach are that it allows direct exposure of internal salient and it does not jeopardize the already compromised pulmonary function. Here the posterior 5 cm of three crowded ribs at apex are removed along with the transverse processes. Care is needed to remain extra pleural or retroperitoneal. Segmental intercostal nerves serve as a guide to intervertebral foramina. Two or three pedicles at apex are removed and dura is seen. One or more intercostal vessels are excised. The pleura is elevated at the apex of kyphosis. The removal of posterior half of the collapsed vertebrae is done with a high speed burr leaving behind posterior rim until last to avoid forward migration of dural sac. This allows spinal cord to be transposed anteriorly and adequate length of anterior dura is exposed [Figure 7]. Cortical strut grafting is performed as far anteriorly as possible after exposing the proximal and distal limits of angular kyphus.

## CONCLUSION

Kyphosis in spinal TB continues to progress on treatment and in children, it increases with growth. Long standing severe kyphosis produces painful costopelvic impingement, reduced vital capacity, lumbar canal stenosis and late onset paraplegia.

The development of kyphosis can be prevented by diagnosing it in pre-destructive stage. The kyphosis should be corrected in the patients who report with severe kyphosis or likely to progress 50° or more on treatment. To correct kyphosis in tubercular spine, anterior decompression and corpectomy, posterior column shortening, posterior instrumentation as well as anterior and posterior bone grafting need to be sequentially performed. Kyphosis correction can be performed by single stage extra pleural anterolateral approach, transpedicular approach or by single stage or two-stage anterior and posterior approaches.
